# Glimepiride reduces CD14 expression and cytokine secretion from macrophages

**DOI:** 10.1186/1742-2094-11-115

**Published:** 2014-06-21

**Authors:** Victoria Ingham, Alun Williams, Clive Bate

**Affiliations:** 1 Department of Pathology and Pathogen Biology, Royal Veterinary College, Hawkshead Lane, North Mymms, Herts, London, UK; 2 Department of Veterinary Medicine, University of Cambridge, Madingley Road, Cambridge CB3 OES, UK

## Abstract

**Background:**

Activated microglia are associated with deposits of aggregated proteins within the brains of patients with Alzheimer’s disease (AD), Parkinson’s disease (PD) and prion diseases. Since the cytokines secreted from activated microglia are thought to contribute to the pathogenesis of these neurodegenerative diseases, compounds that suppress cytokine production have been identified as potential therapeutic targets. CD14 is a glycosylphosphatidylinositol (GPI)- anchored protein that is part of a receptor complex that mediates microglial responses to peptides that accumulate in prion disease (PrP82-146), AD (amyloid-β (Aβ)_42_) and PD (α-synuclein (αSN)). As some GPI-anchored proteins are released from cells by treatment with glimepiride, a sulphonylurea used for the treatment of diabetes, the effects of glimepiride upon CD14 expression and cytokine production from cultured macrophages were studied.

**Methods:**

RAW 264 cells and microglial cells were treated with glimepiride or phosphatidylinositol (PI)-phospholipase C (PLC) and the expression of cell receptors was analysed by ELISA and immunoblot. Treated cells were subsequently incubated with Aβ_42_, αSN, PrP82-146 or lipopolysaccharide (LPS) and the amounts of Toll-like receptor (TLR)-4, tumour necrosis factor (TNF), interleukin (IL)-1 and IL-6 measured.

**Results:**

Glimepiride released CD14 from RAW 264 cells and microglial cells. Pre-treatment with glimepiride significantly reduced TNF, IL-1 and IL-6 secretion from RAW 264 and microglial cells incubated with LPS, Aβ_42_, αSN and PrP82-146. Glimepiride also reduced the LPS, Aβ_42_, αSN and PrP82-146-induced translocation of TLR-4 into membrane rafts that is associated with cell activation. These effects of glimepiride were also seen after digestion of RAW 264 cells with PI-phospholipase C (PLC). In addition, the effects of glimepiride were blocked by pharmacological inhibition of GPI-PLC. The cytokine production was CD14-dependent; it was reduced in microglia from CD14 knockout mice and was blocked by antiserum to CD14.

**Conclusions:**

RAW 264 and microglial cell responses to Aβ_1–42_, αSN, PrP82-146 and LPS are dependent upon CD14 expression. Glimepiride induced the shedding of CD14 from cells by activation of GPI-PLC and consequently reduced cytokine production in response to Aβ_42_, αSN, PrP82-146 and LPS. These results suggest that glimepiride acts as a novel anti-inflammatory agent that could modify the progression of neurodegenerative diseases.

## Background

The deposition of aggregated proteins within the brain is a common feature of neurodegenerative diseases including Alzheimer’s disease (AD), Parkinson’s disease (PD) and prion diseases. These aggregates are often surrounded by activated microglial cells, the resident macrophage-like cells of the brain [1-3] and, *in vitro* aggregated forms of disease-associated peptides stimulate cytokine secretion from microglia/macrophages [3-6]. Numerous studies suggest that cytokine-induced neuroinflammation contributes to the clinical progression of AD, PD and prion diseases [7-9]. For example, epidemiological studies reported that the use of non-steroidal anti-inflammatory drugs delayed the progression of dementia, PD and AD [10-12] and the beneficial effects of statin therapy on dementia [13] have been attributed to their anti-inflammatory properties [14]. While it is difficult to ascertain the extent of cytokine secretion that occurs within the brain directly, the addition of fibrillar forms of amyloid-β (Aβ_1–42_), α-synuclein (αSN) or the prion-derived peptide (PrP82-146) stimulated the secretion of cytokines from cultured macrophages and microglia [3,15-17]. In this study the responses of a macrophage cell line (RAW 264 cells) and primary microglial cells that secrete tumour necrosis factor (TNF) and interleukins (IL) 1 and IL-6 [18] in response to PrP82-146, Aβ_1–42_ and αSN were studied.

It has been suggested that drugs that reduce cytokine secretion from macrophages might be of therapeutic benefit in AD and PD [19]. While multiple receptors have been reported to be involved in macrophage responses to aggregated neurotoxic proteins [20], including scavenger receptors [21] and CD40 [22], other studies implicate CD14, a protein that is highly expressed on myeloid cells including microglia [23], as a key component of the receptor complex that mediates cytokine secretion induced by fibrillar Aβ peptides [16], prion-damaged neurons [24] and lipopolysaccharide (LPS) [25,26]. Furthermore, the genetic deletion of CD14 attenuates pathology in a murine model of AD [27]. We therefore hypothesised that any compound that reduces the expression of CD14 on microglia/macrophages may also reduce cytokine secretion and consequently ameliorate the rate of cognitive decline. Little is known about what factors control the expression of CD14 on macrophages. However, CD14 is linked to the membrane via a glycosylphosphatidylinositol (GPI) anchor [28] and like other GPI-anchored proteins is found in soluble forms. Thus, soluble CD14 found in the bloodstream can reduce cytokine secretion from macrophages [29] and prevent mortality in LPS-treated mice [30]. More recently, the concentrations of soluble CD14 were found to be elevated in AD and PD patients and were associated with glial cell suppression [31]. Collectively such observations suggest that either a reduction of cell-associated CD14, or an increase in soluble CD14 in extracellular fluids, can reduce cytokine secretion from macrophages.

Glimepiride, a sulphonylurea used to treat diabetes [32], is able to mimic insulin signalling and activate an endogenous GPI-phospholipase C (PLC) [33] resulting in the release of some GPI-anchored proteins from the surface of adipocytes [34] and neurons [35]. For this reason, its effects upon macrophages were examined in this study. We report that glimepiride-treated RAW 264 and microglial cells expressed lower amounts of CD14 and produced fewer cytokines when incubated with PrP82-146, Aβ_1–42_, αSN or LPS than control cells.

## Methods

### Cell lines

The murine RAW 264 cell line was grown in Ham’s F12 medium supplemented with 2 mM glutamine, 5% foetal calf serum (FCS) (PAA, http://www.gelifesciences.com/PAA) and standard antibiotics (100 U/ml penicillin and 100 μg/ml streptomycin - Invitrogen, http://www.lifetechnologies.com/uk). Cells were seeded in 48-well plates at 2 x 10^5^ cells per well and allowed to adhere overnight. Cells were pre-treated with test compounds including glimepiride, glipizide, p-chloromercuriphenylsulphonate (p-CMPS), polymyxin B or phosphatidylinositol (PI)-PLC derived from *Bacillus cereus,* (all obtained from Sigma) or with antibodies to CD14 (goat polyclonal IgG anti-mouse CD14 (R&D Systems, http://www.rndsystems.com), PrP^C^ (mouse mAb 4 F2) or CD55 (rat mAb 3D5 Abcam) for one hour before the addition of LPS (Sigma, http://www.sigmaaldrich.com), PrP82-146, αSN or Aβ_42_. Cell supernatants were collected 24 hours later and tested for cytokines or CD14. Cell extracts were also collected and tested for CD14 and Toll-like receptor (TLR)-4. Experiments were performed in triplicate wells. Levels of TNF, IL-1 and IL-6 were assayed by sandwich enzyme-linked immunoassays (ELISA)s using matched pairs of antibodies (see below).

### Microglial cells

These were prepared by dissociating cerebral cortices of newborn mouse pups derived from C57Bl/B6J, CD14 wild type and from C57Bl/B6J, CD14 knockout mice (Jackson Laboratory, http://www.jax.org) as described [36]. Cell suspensions were prepared by repeated passage through a fine bore pipette and debris was removed by filtration through a 100 μM cell strainer. Cells were suspended in Ham’s F12 medium containing 2 mM glutamine, standard antibiotics and 10% FCS, and placed into poly-L-lysine coated 75 cm^3^ flasks. Cultures were maintained at 37°C with 5% CO_2_ for two weeks or until glial cultures were confluent. Microglial cells were isolated by shaking cultures at 260 rpm for 2 hours. Detached cells were collected, counted and dispensed at 2 × 10^5^ cells per well in 48-well plates. Greater than 90% of cells stained positive for the mouse macrophage marker protein F4/80 (Serotec, http://www.abdserotec.com/).

### Cell extracts

At the end of treatments, cells were washed twice in PBS and homogenised in an extraction buffer containing 10 mM Tris-HCl at pH 7.4, 100 mM NaCl, 10 mM ethylenediaminetetraacetic acid (EDTA), 0.5% Nonidet P-40, 0.5% sodium deoxycholate, 0.2% sodium dodecyl sulphate (SDS) and mixed protease inhibitors (4-(2-aminoethyl) benzenesulfonyl fluoride, aprotinin, leupeptin, bestatin, pepstatin A and E-46 - Sigma) at 10^6^ cells/ml. Membranes were prepared by repeated passage with a Wheaton homogeniser; nuclei and large fragments were removed by centrifugation (300 × *g* for 5 minutes).

### Isolation of detergent resistant membranes (DRMs)/lipid rafts

Cells were lysed in an ice-cold buffer containing 1% Triton X-100, 10 mM Tris-HCl (pH 7.2), 100 mM NaCl, 10 mM EDTA and mixed protease inhibitors (10^6^ cells/ml). Membranes were prepared by homogenisation and nuclei and large fragments were removed by centrifugation (300 × *g* for 5 minutes). The post nuclear supernatant was incubated on ice for 60 minutes prior to further centrifugation (16,000 × *g* for 30 minutes at 4°C). The soluble material was reserved as the normal cell membrane. The pellets were solubilised in extraction buffer (10 mM Tris-HCl, 10 mM NaCl, 10 mM EDTA, 0.5% Nonidet P-40, 0.5% sodium deoxycholate and 0.2% SDS and mixed protease inhibitors, centrifuged again (10 minutes at 16,000 × *g*) and the supernatant reserved as the lipid raft fraction.

### IL-1β ELISA

A monoclonal antibody to IL-1β (R&D systems) was diluted to 2 μg/ml in carbonate buffer and dispensed in 96-well Maxisorb Immunoplates (Nunc, Roskilde, Denmark) overnight. Samples were added for one hour, followed by the secondary antibody (a biotinylated goat anti-mouse IL-1β (R&D systems)). Extravidin-alkaline phosphatase (1:1,000, Sigma) was added for 1 hour after which 1 mg/ml 4-nitrophenyl phosphate substrate (Sigma) was added. After 20 minutes, plates were read at 450 nM and results were calculated by reference to a standard curve of recombinant murine IL-1β (R&D systems).

### IL-6 ELISA

A monoclonal antibody to IL-6 was diluted to 2 μg/ml in carbonate buffer and dispensed in 96-well Maxisorb Immunoplates overnight. Samples were added for one hour, followed by the secondary antibody (a polyclonal anti-mouse IL-6 conjugated to alkaline phosphatase (R&D systems)) which was detected using 1 mg/ml 4-nitrophenyl phosphate. Plates were read at 450 nM and results were calculated by reference to a standard curve of recombinant murine IL-6 (R&D systems).

### TNF ELISA

Polyclonal IgG to TNF (R&D systems) was diluted to 2 μg/ml in carbonate buffer and dispensed in 96-well Maxisorb Immunoplates overnight. Samples were added for one hour, followed by the detection antibody, a biotinylated anti-mouse TNF polyclonal antibody (R&D systems) added at 200 ng/ml. A 1:5,000 dilution of extravidin-alkaline phosphatase was then added for a further hour followed by the addition of 1 mg/ml 4-nitrophenyl phosphate. Plates were read at 450 nM and results were calculated by reference to a standard curve of recombinant murine TNF (R&D systems).

### CD14 ELISA

Maxisorb Immunoplates were coated with 0.5 μg/ml rat IgG1 anti-mouse CD14 mAb (clone RmC5-3) (BD Biosciences, http://www.bdbiosciences.com). Samples were applied and the amount of bound CD14 was measured using a goat polyclonal IgG anti-mouse CD14 (R&D Systems), followed by anti-goat IgG conjugated to alkaline phosphatase (Sigma) and 1 mg/ml 4-nitrophenyl phosphate (Sigma). Absorbance was measured on a microplate reader at 405 nm. Results were reported as units CD14 where 100 units = amount of CD14 in 10^6^ control RAW 264 cells.

### TLR-4 ELISA

Maxisorb Immunoplates were coated with 0.5 μg/ml of mouse monoclonal to TLR-4 (Abcam - ab22048) (epitope 100 to 200) and blocked with 5% milk powder. Samples were applied (one hour at room temperature) and the amounts of TLR-4 were measured using a rabbit polyclonal IgG to TLR-4 (Abcam - ab13556) (epitope 420 to 435), followed by anti-rabbit IgG conjugated to alkaline phosphatase and 1 mg/ml 4-nitrophenyl phosphate. Absorbance was measured on a microplate reader at 405 nm. Results were reported as% TLR-4 where 100% = amount of TLR-4 in cell extracts from control RAW 264 cells.

### PrP82-146 ELISA

The amount of PrP82-146 in cell extracts was determined by ELISA as described [35]. Maxisorb Immunoplates were coated with mAb 3 F4 (Abcam, http://www.abcam.com). Samples were boiled with 0.2% SDS to denature peptide and incubated for one hour. Bound PrP82-146 was detected with biotinylated ICSM35 (a gift from Dr Mourad Tayebi), followed by extravidin-alkaline phosphatase and 1 mg/ml 4-nitrophenyl phosphate. Absorbance was measured on a microplate reader at 405 nm and the amount of PrP82-146 in cell extracts was calculated by reference to a standard curve of PrP82-146.

### Aβ_42_ ELISA

Maxisorb Immunoplates were coated with mAb 4G8 (epitope 17 to 24) (Covance, http://www.bioscience.co.uk/covance) in carbonate buffer overnight. Plates were blocked with 5% milk powder in PBS-Tween and samples were applied. The detection antibody was an Aβ_42_ selective rabbit mAb BA3-9 (Covance) followed by biotinylated anti-rabbit IgG (Sigma). Total Aβ was visualised by addition of 1 mg/ml 4-nitrophenyl phosphate and optical density was read in a spectrophotometer at 405 nm. Absorbance was measured on a microplate reader at 405 nm and results were calculated by comparison to serial dilutions of synthetic Aβ_1–42_.

### Western blot analysis

Samples were mixed with Laemmli buffer, heated to 95°C and subjected to electrophoresis on 15% polyacrylamide gels. Proteins were transferred onto a Hybond-P PVDF membrane (Amersham Biotech, http://www.gelifesciences.com) by semi-dry blotting. Membranes were blocked with 10% milk powder and incubated with either mAb ICSM35 (to detect PrP^C^), goat polyclonal IgG anti-mouse CD14 (R&D Systems), sheep polyclonal anti-CD55 (R&D Systems) or with rabbit polyclonal antibodies to caveolin (Upstate, http://www.millipore.com), followed by a secondary anti-mouse, rabbit or goat IgG conjugated to peroxidase (Sigma). Bound antibody was visualised using enhanced chemiluminescence.

### Peptides

Synthetic peptides containing amino acids 82 to 146 of the human PrP protein (PrP82-146) corresponding to a PrP fragment found in prion-infected brains, and a control peptide (PrP82-146scrambled) were supplied by Professor Mario Salmona (Mario Negri Institute, Milan). A synthetic peptide containing the amino acid residues 1 to 42 of the Aβ protein (Aβ_1–42_) and a control peptide consisting of the same amino acids in reverse order (Aβ_42–1_) were obtained from Bachem. Stock solutions of peptides were stored at 1 mM in di-methyl sulphoxide and thawed on the day of use before dilution in culture medium, sonication and addition to cells. Recombinant αSN and βSN were purchased from Sigma.

### Statistical methods

Differences between treatment groups were assessed using Student’s, two sample, paired-*t* tests. In all tests statistical significance was set at the 5% level.

## Results

### Glimepiride stimulated the release of CD14 from RAW 264 cells

**Figure 1:**
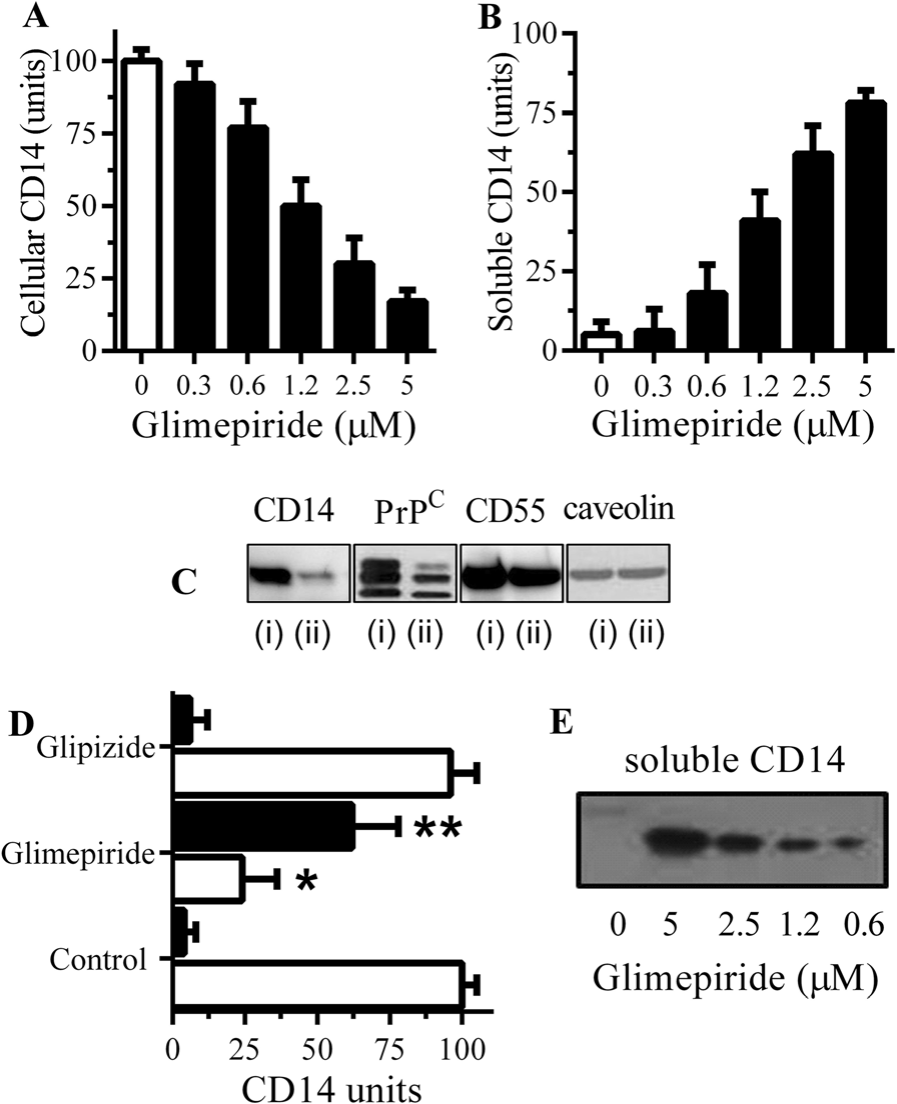
**Glimepiride releases CD14 from RAW 264 cells. (A)** The amounts of CD14 in RAW 264 cells treated for one hour with control medium (□) or glimepiride (■) as shown. Values are means ± SD, from triplicate experiments performed 4 times, n = 12. **(B)** The amounts of CD14 in supernatants from RAW 264 cells treated for one hour with control medium (□) or glimepiride as shown (■). Values are means ± SD, from triplicate experiments performed 4 times, n = 12. **(C)** Immunoblots showing the amounts of CD14, PrP^C^, CD55 and caveolin in extracts from RAW 264 cells treated for 1 hour with control medium (i) or 5 μM glimepiride (ii). **(D)** The amounts of CD14 in cells (□) or supernatants (■) from microglial cells treated for 1 hour with control medium, 5 μM glimepiride or 5 μM glipizide. Values are mean units CD14 ± SD, from triplicate experiments performed 3 times, n = 9. *Cellular CD14 significantly less than control cells. **supernatant CD14 significantly greater than control supernatants. **(E)** Blot showing the amounts of CD14 in supernatants from microglial cells treated with concentrations of glimepiride as shown for one hour.

Since glimepiride stimulated the release of some GPI-anchored proteins from adipocytes [34] and neurons [35] the effect of glimepiride on the expression of GPI-anchored proteins in RAW 264 cells was examined. The amount of CD14 in RAW 264 cells treated with glimepiride for one hour was reduced in a dose-dependent manner (Figure 1A); the loss of CD14 from RAW 264 cells was accompanied by a corresponding increase in the amount of CD14 in the supernatant of these cells (Figure 1B). These effects occurred at non-toxic concentrations of glimepiride, thus the addition of 5 μM glimepiride did not significantly alter cell survival as measured by thiazolyl blue tetrazolium (97% cell viability ± 6 compared with 100% ± 7, n = 12, *P* = 0.24). Pre-treatment of RAW 264 cells with 5 μM glipizide, another sulphonylurea used to treat diabetes, did not affect the amounts of CD14 in cells (data not shown). The effects of glimepiride were not CD14 specific; it also reduced the amount of the GPI-anchored cellular prion protein (PrP^C^) in cells, but did not affect the amounts of GPI-anchored CD55 or caveolin (Figure 1C). Glimepiride, but not glipizide, had a similar effect upon microglial cells; treatment with 5 μM glimepiride reduced cellular CD14 and increased the amounts of soluble CD14 in the supernatant (Figure 1D). Glimepiride caused a dose-dependent increase in the amounts of soluble CD14 as measured by Western blot (Figure 1E).

### Glimepiride reduced cytokine secretion from RAW 264 cells

**Figure 2:**
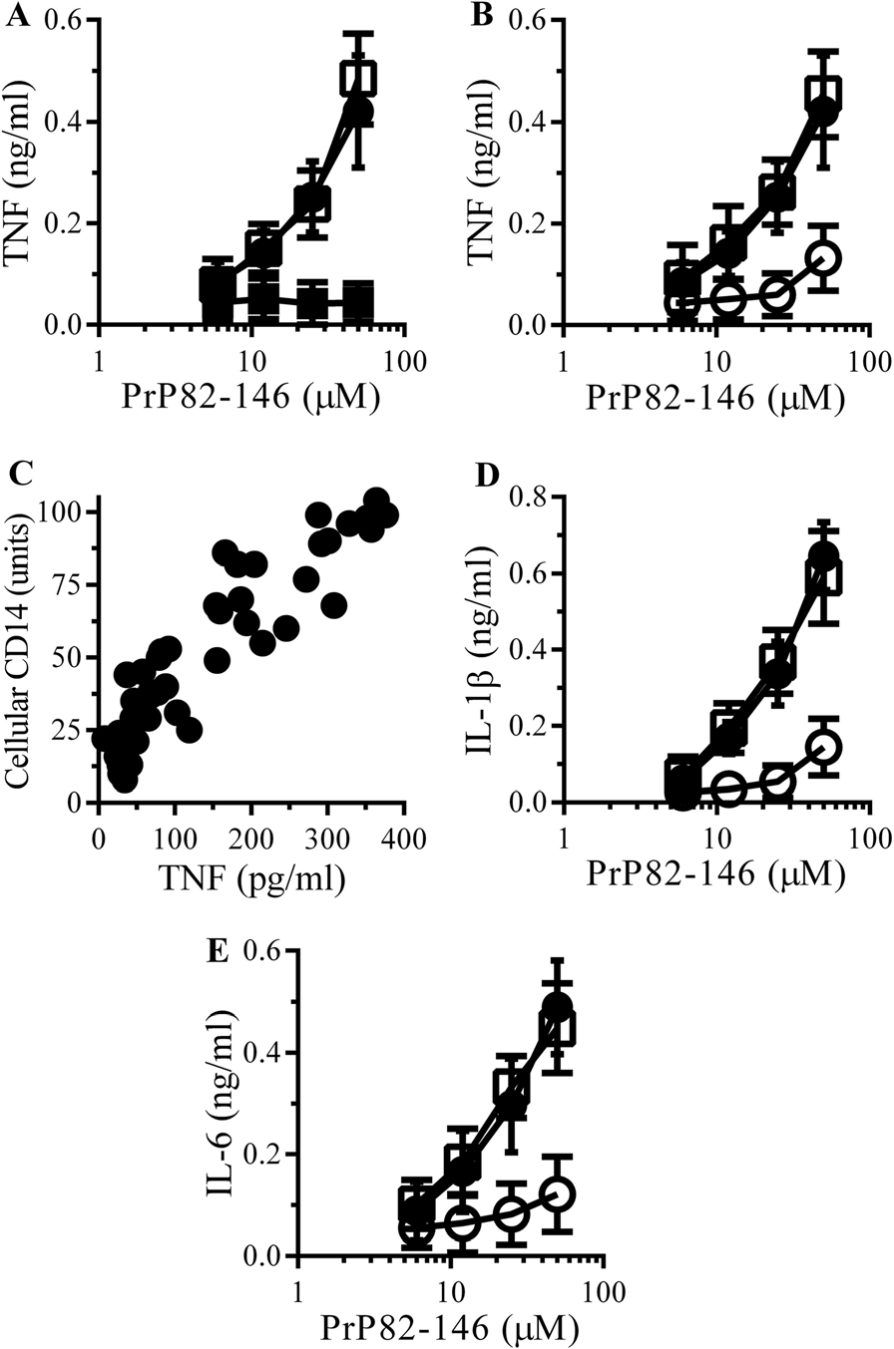
**Glimepiride reduces cytokine secretion from RAW 264 cells incubated with PrP82-146. (A)** The concentrations of TNF produced by RAW 264 cells incubated with PrP82-146 (●), PrP82-146scrambled (■) or PrP82-146 and 1 μg/ml polymyxin B (□). Values are means ± SD from triplicate experiments performed 4 times, n = 12. **(B)** The concentrations of TNF produced by RAW 264 cells pre-treated with control medium (●), 5 μM glimepiride (○) or 5 μM glipizide (□) and incubated with PrP82-146. Values are means ± SD from triplicate experiments performed 4 times, n = 12. **(C)** There was a significant correlation between the amounts of cellular CD14 of RAW 264 cells treated with glimepiride (0.3 to 5 μM) for 1 hour and TNF production after the addition of 50 μM PrP82-146, Pearson’s coefficient = 0.858, *P* < 0.01. **(D)** The concentrations of IL-1β produced by RAW 264 cells pre-treated with control medium (●), 5 μM glimepiride (○) or 5 μM glipizide (□) and incubated with PrP82-146. Values are means ± SD from triplicate experiments performed 4 times, n = 12. **(E)** The concentrations of IL-6 produced by RAW 264 cells pre-treated with control medium (●), 5 μM glimepiride (○) or 5 μM glipizide (□) and incubated with PrP82-146. Values are means ± SD from triplicate experiments performed 4 times, n = 12.

The activation of microglial cells by aggregated peptides is thought to affect the progression of prion diseases. The addition of PrP82-146 increased the secretion of TNF from RAW 264 cells (Figure 2A). TNF production from cells incubated with a control peptide (PrP82-146scrambled) was not significantly different from cells treated with control medium. The inclusion of 1 μg/ml polymyxin B, an antibiotic that blocks the effects of LPS, did not affect PrP82-146-induced TNF production, indicating that cytokine production was not due to contamination with this common bacterial contaminant. The effects of glimepiride on TNF secretion were then tested. The addition of 5 μM glimepiride or 5 μM glipizide did not increase TNF production over that of control cells. However, pre-treatment with 5 μM glimepiride, but not 5 μM glipizide, significantly reduced the secretion of TNF from RAW 264 cells incubated with PrP82-146 (Figure 2B). The effects of glimepiride upon TNF production were dose-dependent; a significant correlation was observed between the amounts of cellular CD14 and TNF production following the addition of 50 μM PrP82-146 (Figure 2C). Similarly, pre-treatment of RAW 264 cells with 5 μM glimepiride, but not glipizide, significantly reduced the secretion of IL-1β (Figure 2D) or IL-6 (Figure 2E).

**Figure 3:**
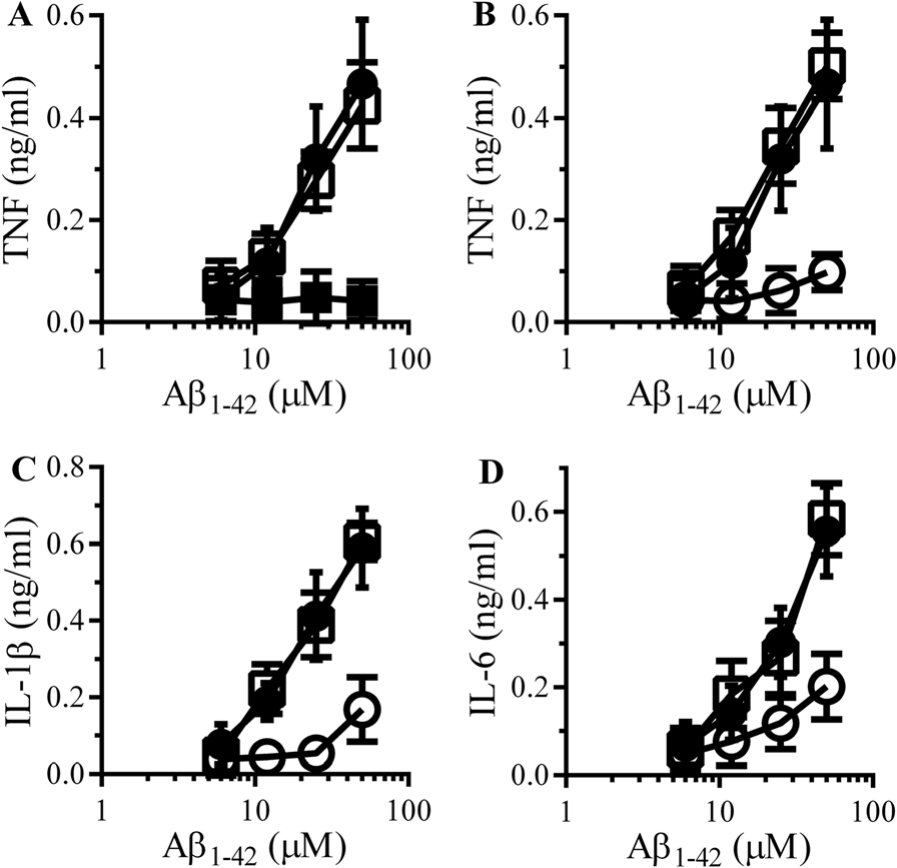
**Glimepiride reduces cytokine secretion from RAW 264 cells incubated with Aβ. (A)** The concentrations of TNF produced by RAW 264 cells incubated with Aβ_1–42_ (●), Aβ_42–1_ (■) or Aβ_1–42_ and 1 μg/ml polymyxin B (□). Values are means ± SD from triplicate experiments performed 4 times, n = 12. **(B)** The concentrations of TNF produced by RAW 264 cells pre-treated with control medium (●) 5 μM glimepiride (○) or 5 μM glipizide (□) and incubated with Aβ_1–42_. Values are means ± SD from triplicate experiments performed 4 times, n = 12. **(C)** The concentrations of IL-1β produced by RAW 264 cells pre-treated with control medium (●) 5 μM glimepiride (○) or 5 μM glipizide (□) and incubated with Aβ_1–42_. Values are means ± SD from triplicate experiments performed 4 times, n = 12. **(D)** The concentrations of IL-6 produced by RAW 264 cells pre-treated with control medium (●), 5 μM glimepiride (○) or 5 μM glipizide (□) and incubated with Aβ_1–42_. Values are means ± SD from triplicate experiments performed 4 times, n = 12.

**Figure 4:**
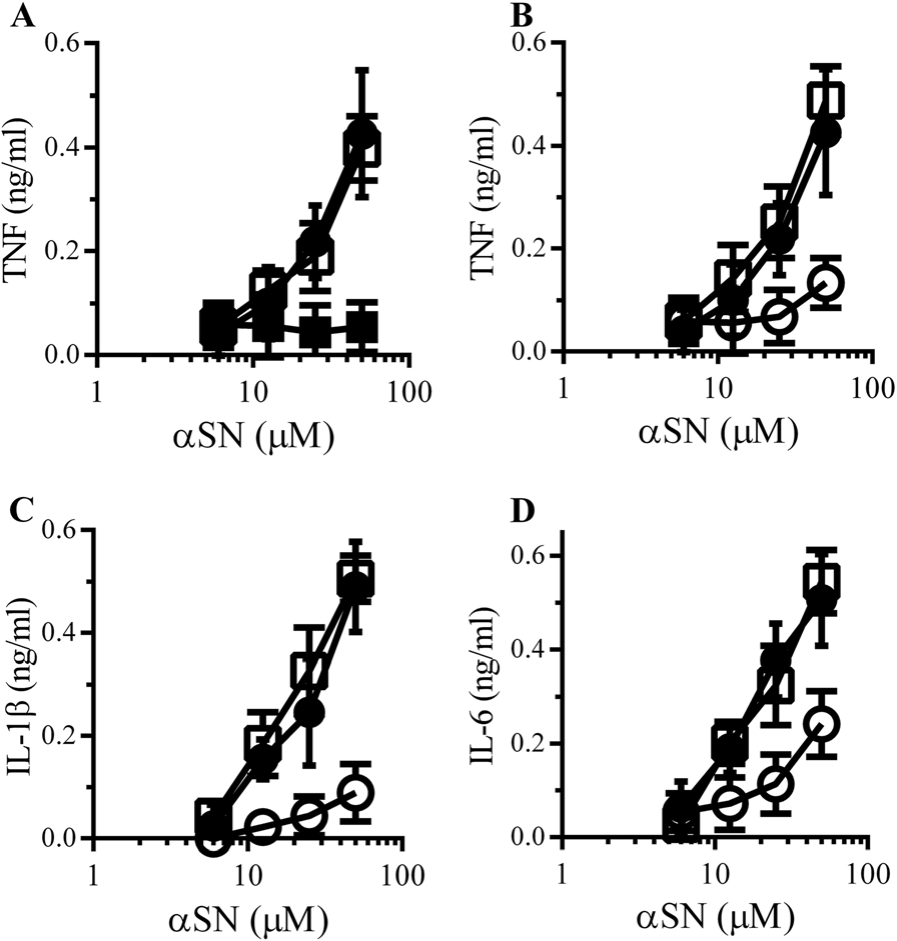
**Glimepiride reduces cytokine secretion from RAW 264 cells incubated with αSN. (A)** The concentrations of TNF produced by RAW 264 cells incubated with αSN (●), βSN (■) or αSN and 1 μg/ml polymyxin B (□). Values are means ± SD from triplicate experiments performed 4 times, n = 12. **(B)** The concentrations of TNF produced by RAW 264 cells pre-treated with control medium (●), 5 μM glimepiride (○) or 5 μM glipizide (□) and incubated with αSN. Values are means ± SD from triplicate experiments performed 4 times, n = 12. **(C)** The concentrations of IL-1β produced by RAW 264 cells pre-treated with control medium (●) or 5 μM glimepiride (○) or 5 μM glipizide (□) and incubated with αSN. Values are means ± SD from triplicate experiments performed 4 times, n = 12. **(D)** The concentrations of IL-6 produced by RAW 264 cells pre-treated with control medium (●), 5 μM glimepiride (○) or 5 μM glipizide (□) and incubated with αSN. Values are means ± SD from triplicate experiments performed 4 times, n = 12.

The addition of Aβ_1–42_, but not Aβ_42–1_, also caused the secretion of TNF from RAW 264 cells (Figure 3A). The Aβ_1–42_-induced secretion of TNF was not affected by the inclusion of 1 μg/ml polymyxin B. Pre-treatment with 5 μM glimepiride, but not 5 μM glipizide, significantly reduced the Aβ_1–42_-induced secretion of TNF (Figure 3B), IL-1β (Figure 3C) or IL-6 (Figure 3D). Similarly, the addition of αSN, but not βSN, caused the secretion of TNF from RAW 264 cells (Figure 4A). The αSN-induced secretion of TNF was not affected by the inclusion of 1 μg/ml polymyxin B. Pre-treatment with 5 μM glimepiride, but not 5 μM glipizide, significantly reduced the αSN-induced secretion of TNF (Figure 4B), IL-1β (Figure 4C) or IL-6 (Figure 4D) from RAW 264 cells.

### Glimepiride reduced cytokine secretion from microglial cells

**Figure 5:**
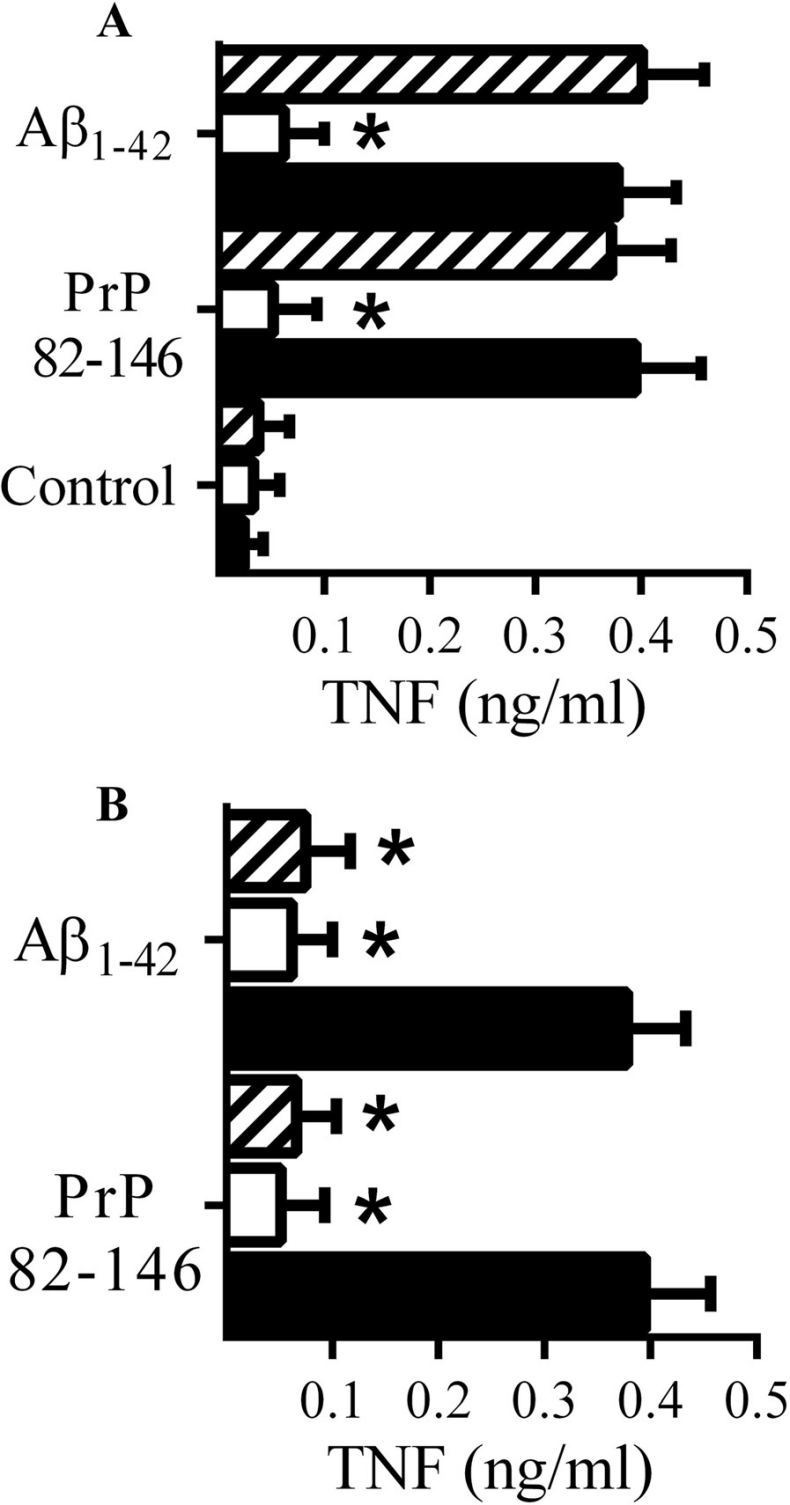
**Glimepiride reduces cytokine secretion from microglial cells incubated with PrP82-146 or Aβ**_**1–42**_**. (A)** The concentrations of TNF produced by microglial cells pre-treated for 1 hour with control medium (■), 5 μM glimepiride (□) or 5 μM glipizide (striped bars) and incubated with control medium, 50 μM PrP82-146 or 50 μM Aβ_1–42_ as shown. Values are means ± SD from triplicate experiments performed 3 times, n = 9. *TNF significantly less than control cells incubated with peptides. **(B)** The concentrations of TNF produced by microglial cells pre-treated with control medium (●) or 5 μM glimepiride for 1 hour (□), or pre-treated with 5 μM glimepirde for 1 hour and washed 3 times with PBS (striped bars) and incubated with 50 μM PrP82-146 or 50 μM Aβ_1–42_ as shown. Values are means ± SD from triplicate experiments performed 3 times, n = 9. *TNF significantly less than control cells incubated with peptides.

Responses from RAW 264 cells were compared to those obtained with primary microglial cells which produced similar amounts of TNF when incubated with either 50 μM PrP82-146 or 50 μM Aβ_1–42_. The amounts of TNF secreted from microglial cells incubated with control peptides, PrP82-146scrambled or Aβ_42–1_, were not significantly higher than those from control microglial cells (data not shown). Pre-treatment of microglial cells with 5 μM glimepiride, but not 5 μM glipizide, significantly reduced TNF secretion in response to 50 μM PrP82-146 or 50 μM Aβ_1–42_ (Figure 5A). It was not clear whether soluble CD14 released from glimepiride-treated cells bound to PrP and Aβ peptides, thus preventing their interaction with microglial cells, or whether glimepiride caused microglial cells to be hyporesponsive to PrP and Aβ peptides. Therefore, microglial cells were treated with 5 μM glimepiride for 1 hour and then thoroughly washed to remove any soluble receptors before the addition of 50 μM PrP82-146 or 50 μM Aβ_1–42._ We report that washed cells produced less TNF than control cells when incubated with 50 μM PrP82-146 or 50 μM Aβ_1–42_ (Figure 5B).

### Immunosuppressive effects of glimepiride are transient

**Figure 6:**
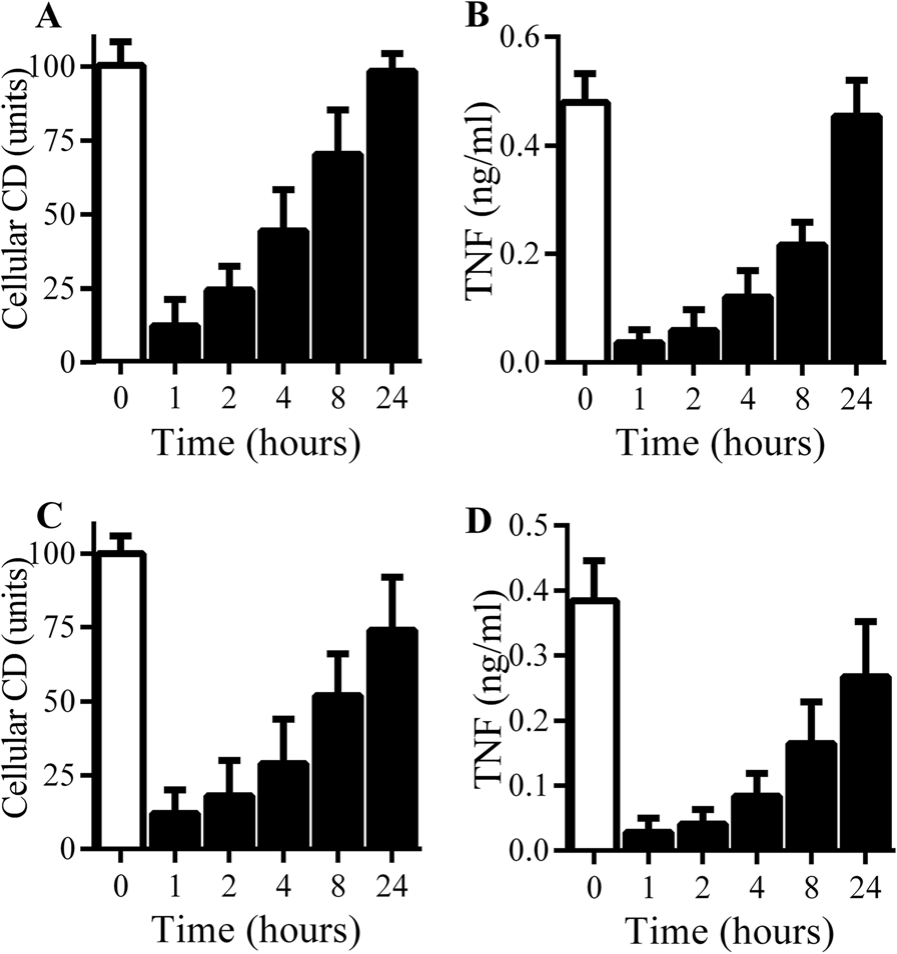
**RAW264 and microglial cells recover from glimepiride-induced suppression of cytokine production. (A)** The amounts of CD14 in RAW 264 cells treated with control medium (□) or 5 μM glimepiride for time periods as shown (■). Values are means ± SD from triplicate experiments performed twice, n = 6. **(B)** The concentrations of TNF produced by RAW 264 cells pre-treated with control medium (□) or 5 μM glimepiride for time periods as shown (■) and incubated with 50 μM PrP82-146 for 24 hours. Values are means ± SD from triplicate experiments performed twice, n = 6. **(C)** The amounts of CD14 in microglial cells treated with control medium (□) or 5 μM glimepiride for time periods as shown (■). Values are means ± SD from triplicate experiments performed twice, n = 6. **(D)** The concentrations of TNF produced by microglial cells pre-treated with control medium (□) or 5 μM glimepiride for time periods as shown (■) and incubated with 50 μM PrP82-146 for 24 hours. Values are means ± SD from triplicate experiments performed twice, n = 6.

A time course study was used to determine whether the effects of glimepiride on RAW 264 and microglial cells were permanent. After treatment with 5 μM glimepiride, there was a time-dependent increase in cell-associated CD14 in both RAW 264 cells (Figure 6A) and microglial cells (Figure 6C). Similarly, the capacity of RAW 264 and microglial cells to produce TNF in response to PrP82-146 also showed a time-dependent increase (Figures 6B and D).

### Glimepiride reduced LPS-induced cytokine secretion

**Figure 7:**
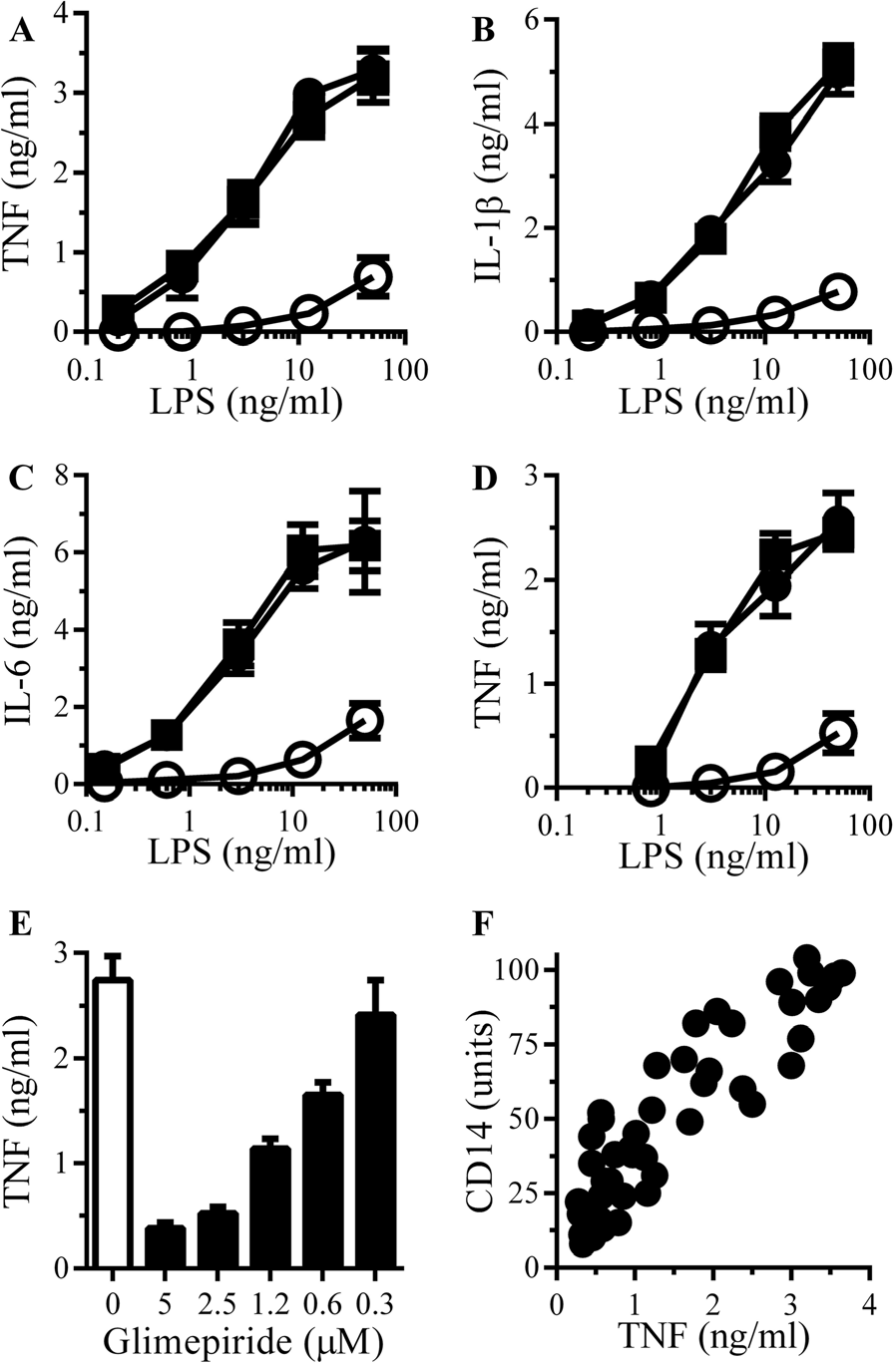
**Glimepiride reduces lipopolysaccharide (LPS)-induced cytokine secretion. (A)** The concentrations of TNF produced by RAW 264 cells pre-treated with control medium (●), 5 μM glimepiride (○) or 5 μM glipizide (■) and incubated with LPS. Values are means ± SD from triplicate experiments performed 4 times, n = 12. **(B)** The concentrations of IL-1β produced by RAW 264 cells pre-treated with control medium (●), 5 μM glimepiride (○) or 5 μM glipizide (■) and incubated with LPS. Values are means ± SD from triplicate experiments performed 4 times, n = 12. **(C)** The concentrations of IL-6 produced by RAW 264 cells pre-treated with control medium (●), 5 μM glimepiride (○) or 5 μM glipizide (■) and incubated with LPS. Values are means ± SD from triplicate experiments performed 4 times, n = 12. **(D)** The concentrations of TNF produced by microglial cells pre-treated with control medium (●), 5 μM glimepiride (○) or 5 μM glipizide (■) and incubated with LPS. Values are means ± SD from triplicate experiments performed 4 times, n = 12. **(E)** The concentrations of TNF produced by RAW 264 cells pre-treated with control medium (□) or glimepiride as shown (■) and incubated with 10 ng/ml LPS. Values are means ± SD from triplicate experiments performed 3 times, n = 9. **(F)** There was a significant correlation between the CD14 content of RAW 264 cells treated with glimepiride (0.3 to 5 μM) and the concentrations of TNF produced in response to 10 ng/ml LPS, Pearson’s coefficient = 0.886.

The effect of glimepiride on cytokine secretion from RAW 264 cells incubated with LPS was also examined. The addition of LPS caused a dose-dependent increase in the secretion of TNF, IL-1β and IL-6 which was reduced by pre-treatment with 5 μM glimepiride (Figure 7A, B and C). In contrast, cytokine secretion was not affected by pre-treatment with 5 μM glipizide. Similarly, pre-treatment with 5 μM glimepiride, but not 5 μM glipizide, reduced TNF production from primary microglial cells incubated with LPS (Figure 7D). When RAW 264 cells were pre-treated with different concentrations of glimepiride before the addition of 10 ng/ml LPS, glimepiride reduced TNF secretion in a dose-dependent manner (Figure 7E). When concentrations of cellular CD14 after treatment with glimepiride (0.3 to 5 μM) were plotted against TNF production in response to LPS, a significant correlation was observed (Figure 7F).

### PI-PLC digestion reduced cytokine secretion from RAW 264 cells

**Figure 8:**
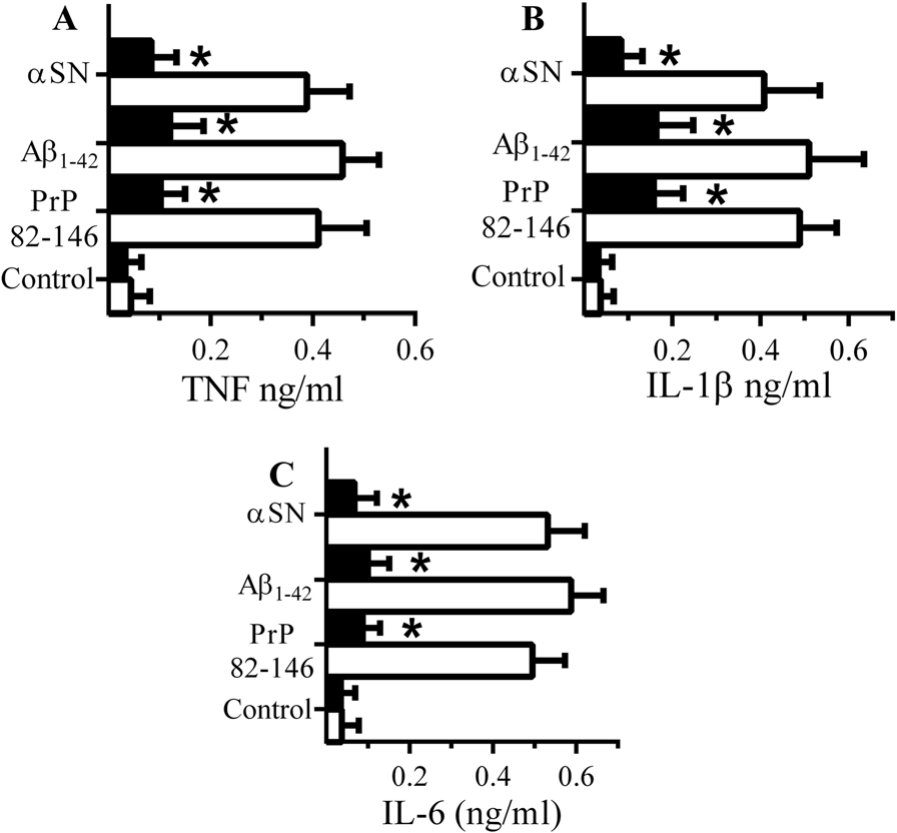
**PI-PLC digestion reduced cytokine secretion from RAW 264 cells. (A)** The concentrations of TNF produced by RAW 264 cells pre-treated with control medium (□) or PI-PLC (■) and incubated with 50 μM PrP82-146, Aβ_1–42_ or αSN. Values are means ± SD from triplicate experiments performed 4 times, n = 12. *TNF significantly less than control cells incubated with peptides. **(B)** The concentrations of IL-1β produced by RAW 264 cells pre-treated with control medium (□) or PI-PLC (■) and incubated with 50 μM PrP82-146, Aβ_1–42_ or αSN. Values are means ± SD from duplicate experiments performed 4 times, n = 8. *IL-1β significantly less than control cells incubated with peptides. **(C)** The concentrations of IL-6 produced by RAW 264 cells pre-treated with control medium (□) or PI-PLC (■) and incubated with 50 μM PrP82-146, Aβ_1–42_ or αSN. Values are means ± SD from duplicate experiments performed 4 times, n = 8. *IL-6 significantly less than control cells incubated with peptides. *IL-6 significantly less than control cells incubated with peptides.

**Figure 9:**
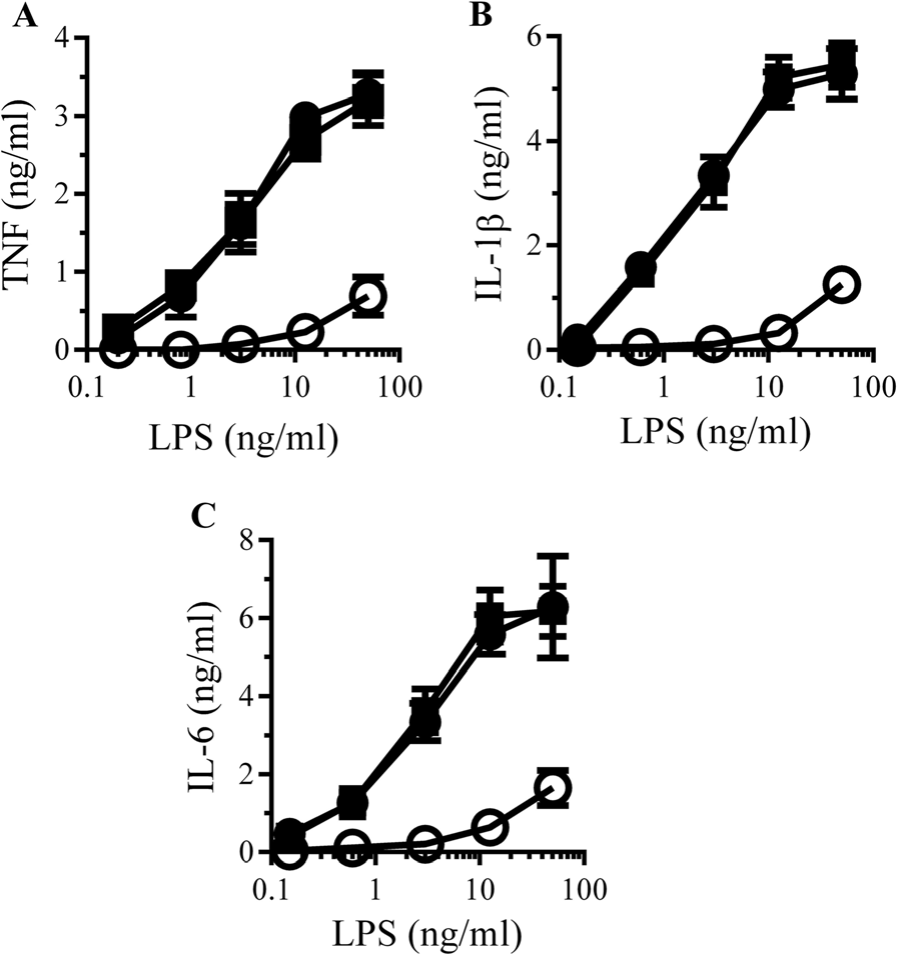
**RAW 264 cells digested with PI-PLC are hyporesponsive to lipopolysaccharide (LPS). (A)** The concentrations of TNF produced by RAW 264 cells pre-treated with control medium (●), PI-PLC (○) or heat-inactivated PI-PLC (■) and incubated with LPS. Values are means ± SD from triplicate experiments performed 4 times, n = 12. (B) The concentrations of IL-1β produced by RAW 264 cells pre-treated with control medium (●), PI-PLC (○) or heat-inactivated PI-PLC (■) and incubated with LPS. Values are means ± SD from triplicate experiments performed 4 times, n = 12. **(C)** The concentrations of IL-6 produced by RAW 264 cells pre-treated with control medium (●), PI-PLC (○) or heat-inactivated PI-PLC (■) and incubated with LPS. Values are means ± SD from triplicate experiments performed 4 times, n = 12.

The molecular mechanisms of glimepiride-induced immunosuppression were examined. Since glimepiride activates an endogenous GPI-PLC causing the release of GPI-anchored proteins [34,35], we hypothesised that treatment with PI-PLC would have similar effects to glimepiride on macrophages. Treatment of RAW 264 cells with 0.2 units PI-PLC/ml for 1 hour significantly reduced the amount of cell associated CD14 (7 units ± 3 compared with 100 ± 10, n = 9, *P* < 0.05) and increased the amount of CD14 in cell supernatants (81 units ± 6 compared with 7 units ± 4, n = 9, *P* < 0.05), thus replicating some of the effects of glimepiride upon RAW 264 cells. Critically, RAW 264 cells pre-treated with of 0.2 units/ml PI-PLC for 1 hour produced significantly less TNF, Il-1β and IL-6 when incubated with 50 μM PrP82-146, Aβ_1–42_ or αSN than did vehicle-treated cells (Figure 8A, B and C). These cells also produced less TNF, IL-1β and IL-6 in response to LPS (Figure 9A, B and C).

**Figure 10:**
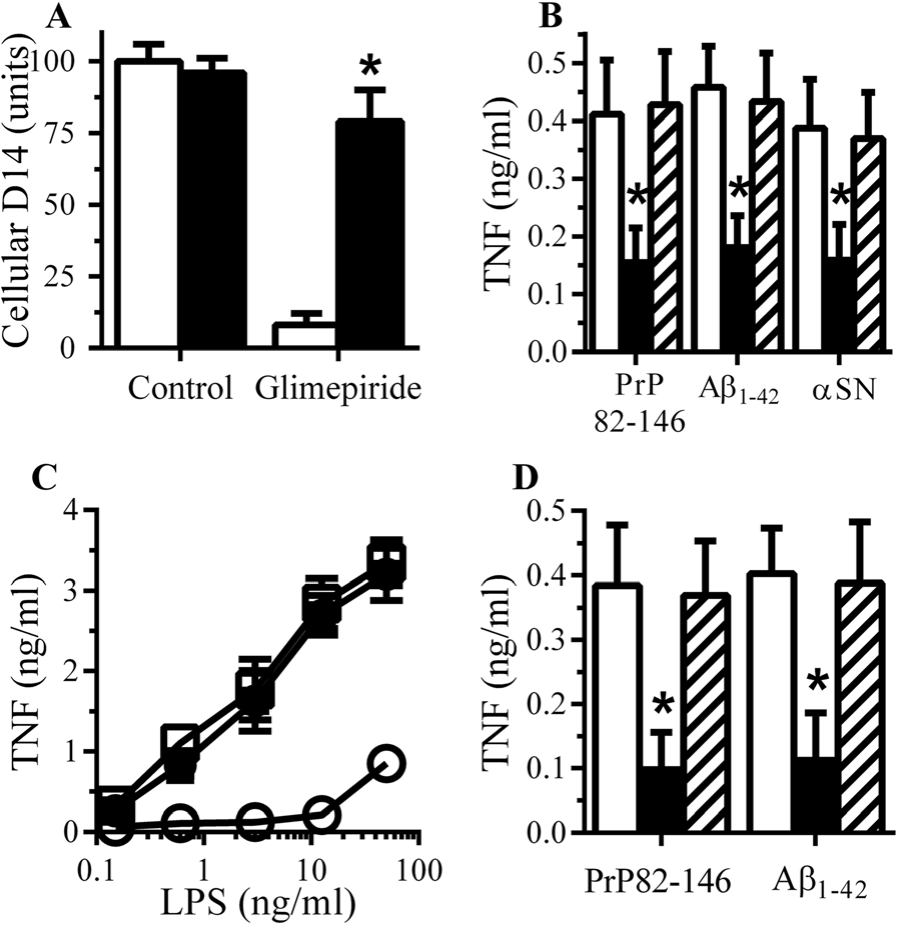
**Glimepiride induced immunosuppression is reversed by pCMPS. (A)** The amounts of CD14 in RAW 264 cells treated for 1 hour with control medium or 5 μM glimepiride in the presence of control medium (□) or 200 μM pCMPS (■). Values are means ± SD from triplicate experiments performed 4 times, n = 12. *CD14 significantly greater than those of cells treated with glimepiride. **(B)** The concentrations of TNF produced by RAW 264 cells pre-treated with control medium (□), 5 μM glimepiride (■), or 5 μM glimepiride and 200 μM pCMPS (striped bars) and incubated with 50 μM PrP82-146, 50 μM Aβ_1–42_ or 50 μM αSN. Values are means ± SD from triplicate experiments performed 4 times, n = 12. *TNF significantly less than control cells incubated with peptides. **(C)** The concentrations of TNF produced by RAW 264 cells pre-treated with control medium (●), 5 μM glimepiride (○) or 5 μM glimepiride and 200 μM pCMPS (*■*) and incubated with LPS as shown. Values are means ± SD from triplicate experiments performed 4 times, n = 12. **(D)** The concentrations of TNF produced by microglial cells pre-treated with control medium (□), 5 μM glimepiride (■), or 5 μM glimepiride and 200 μM pCMPS (striped bars) and incubated with 50 μM PrP82-146 or 50 μM Aβ_1–42_. Values are means ± SD from triplicate experiments performed twice, n = 6. *TNF significantly less than control cells incubated with peptides.

Next, we sought to reverse the effect of glimepiride by inhibiting the activity of GPI-PLC. The effects of pCMPS (an inhibitor of GPI-PLC [37]) upon glimepiride-induced suppression of cytokine production were studied. Treatment with 200 μM p-CMPS did not affect the amount of CD14 in RAW 264 cells (94 units ± 11 compared with 100 ± 8, n = 9, *P* = 0.5), nor did it affect TNF secretion from RAW 264 cells incubated with 10 ng/ml LPS (2,614 pg/ml ± 338 compared with 2,549 pg/ml ± 306, n = 9, *P* = 0.7). However, the addition of 200 μM p-CMPS to RAW 264 cells reversed the effect of glimepiride on cells, it prevented the glimepiride-induced reduction in cellular CD14 (Figure 10A) and reduced the glimepiride-induced suppression of TNF secretion in response to PrP82-146, Aβ_42_ and αSN (Figure 10B) or LPS (Figure 10C). Primary microglial cells behaved in a similar manner to the RAW 264 cells in that the glimepiride-induced suppression of PrP82-146 and Aβ_42_-TNF production was reversed by the inclusion of 200 μM p-CMPS (Figure 10D).

### CD14 is involved in peptide-induced cytokine secretion

**Figure 11:**
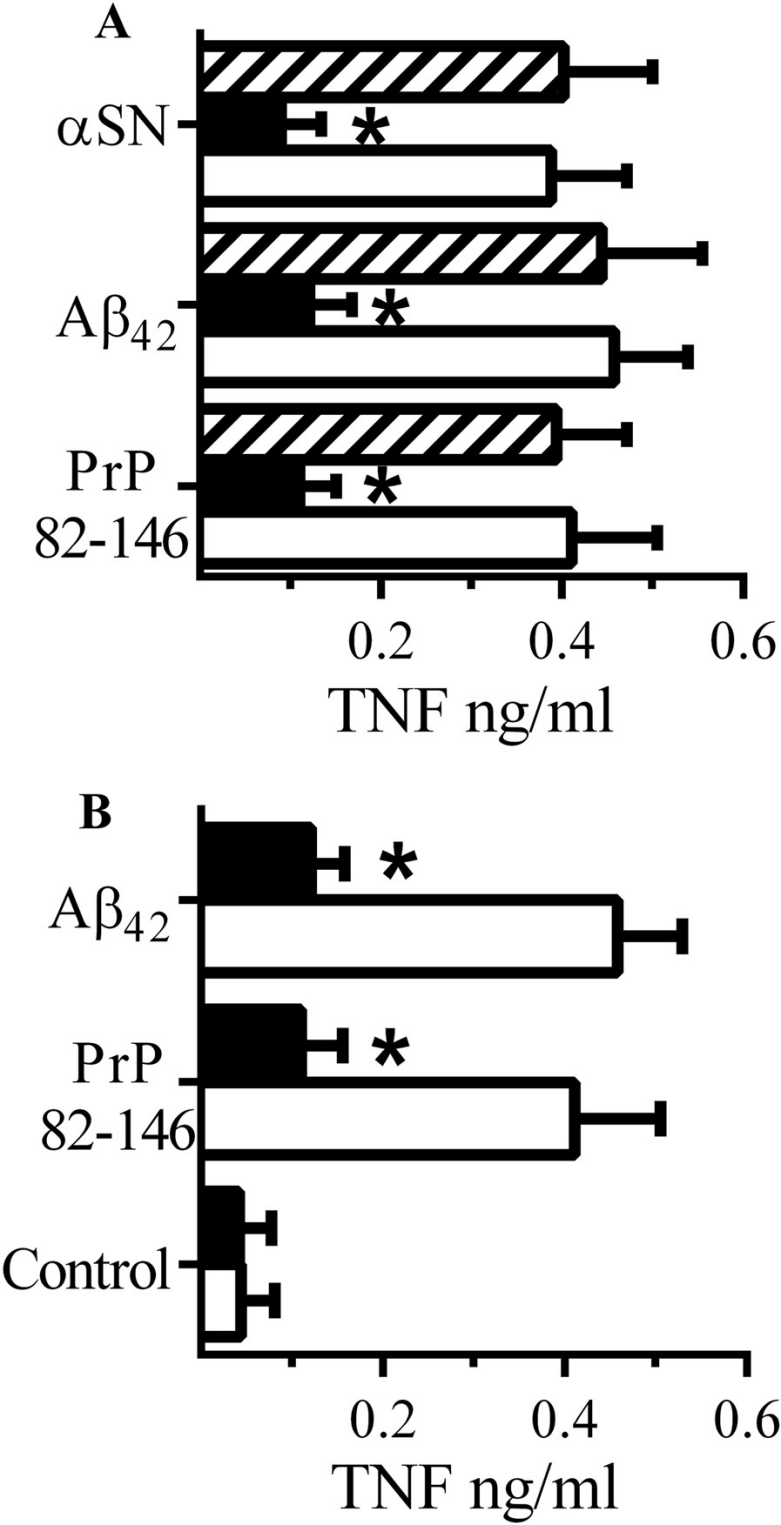
**CD14 mediates cytokine secretion from peptide stimulated RAW 264 cells. (A)** The concentrations of TNF produced by RAW 264 cells pre-treated with antiserum to CD14 (■), PrP^C^ (□) or CD55 (striped bars) and incubated with 50 μM PrP82-146, Aβ_1–42_ or αSN. Values are means ± SD from triplicate experiments performed 3 times, n = 9. *TNF significantly less than those of control cells incubated with peptides. **(B)** The concentrations of TNF produced by microglial cells derived from CD14 wild type (□) or CD14 knockout (■) mice incubated with PrP82-146 or Aβ_1–42_. Values are means ± SD from triplicate experiments performed 3 times, n = 9. *TNF significantly less than those from CD14 wild type cells incubated with peptides.

These results are consistent with the hypothesis that the inhibitory effects of glimepiride on cytokine secretion were mediated through a GPI-anchored protein, and while CD14 is known to be involved in LPS-induced cytokine secretion [25], other GPI-anchored proteins such as the cellular prion protein (PrP^C^) and CD55 may also be involved in the activation of macrophages [26,38]. To determine which of these receptors were critical for peptide-induced cytokine production RAW 264 cells were pre-treated with antibodies to CD14, PrP^C^ or CD55. Pre-treatment of RAW 264 cells with antiserum to CD14 but not by antiserum to PrP^C^ or to CD55 reduced TNF secretion from RAW 264 cells incubated with PrP82-146, Aβ_42_ or αSN (Figure 11A) indicating that CD14 is a major receptor involved in peptide-induced TNF secretion. This hypothesis was strengthened by observations that microglial cells derived from CD14 knockout mice produced less TNF than microglial cells derived from CD14 wild type mice when incubated with PrP82-146 or Aβ_42_ (Figure 11B). Notably, TNF secretion from microglial cells derived from PrP^C^ knockout mice was not significantly different from microglia derived from Prnp wild type mice when incubated with PrP82-146 or Aβ_42_ (data not shown).

Our results suggest that the immunosuppressant effects of glimepiride were mediated by the reduced expression of CD14, a molecule that acts as a receptor for LPS and neurotoxic peptides associated with prion, Alzheimer’s disease and Parkinson’s disease. To determine if glimepiride reduced the uptake of neurotoxic peptides to RAW 264 cells, glimepiride-treated cells were incubated with 50 μM PrP82-146 or 50 μM Aβ_42_ for 2 hours. Pre-treatment with 5 μM glimepiride did not significantly alter the concentration of PrP82-146 (43 μM ± 5 compared with 41 μM ± 7, n = 9, *P* = 0.5) or Aβ_42_ (39 μM ± 8 compared with 40 μM ± 7, n = 9, *P* = 0.7) in RAW 264 cells.

### Peptides cause the translocation of TLR-4 into membrane rafts

**Figure 12:**
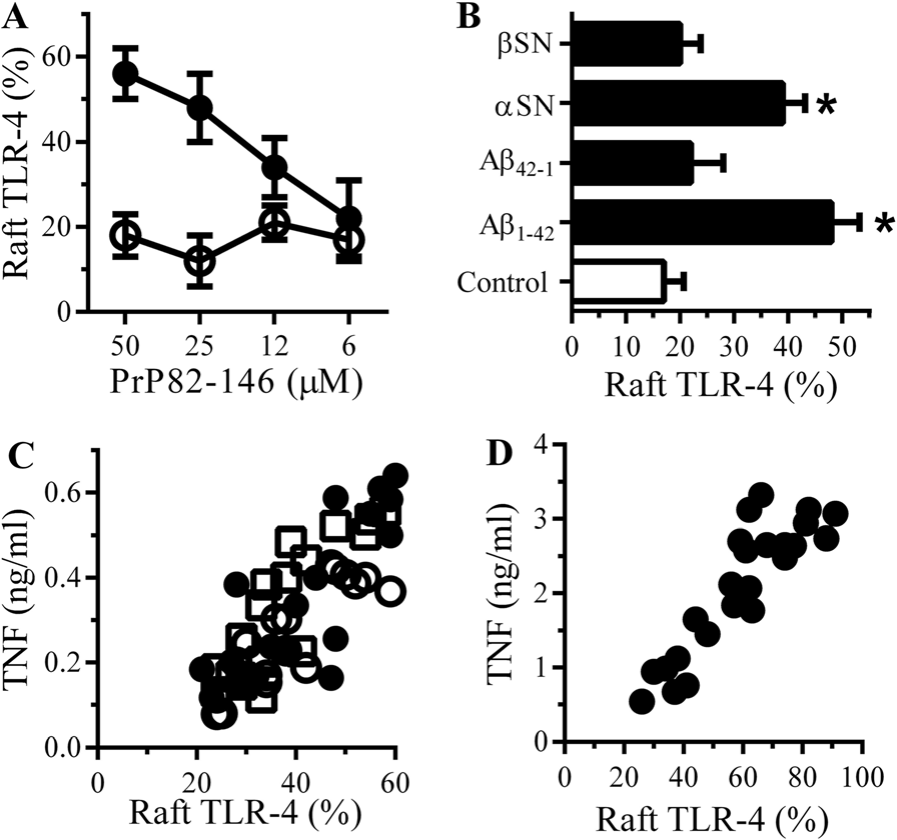
**PrP82-146 causes the translocation of Toll-like receptor (TLR)-4 into rafts. (A)** The % of TLR-4 in DRMs derived from RAW 264 cells treated with PrP82-146 (●) or PrP82-146scrambled (○). Values are means ± SD from triplicate experiments performed 3 times, n = 9. **(B)** The % TLR-4 in DRMs derived from RAW 264 cells treated with control medium (□) or 50 μM Aβ_1–42_, Aβ_42–1_, αSN or βSN as shown (■). Values are means ± SD from triplicate experiments performed 3 times, n = 9. *TLR-4 significantly greater than those of control cells. **(C)** RAW 264 cells were incubated with PrP82-146 (○), Aβ_1–42_ (●) or αSN (□) (6 to 50 μM). There were significant correlations between % TLR-4 in rafts and TNF for PrP82-146, Pearson’s coefficient = 0.88, Aβ_1–42_, Pearson’s coefficient = 0.78 and αSN Pearson’s coefficient = 0.86. **(D)** There was a significant correlation between the % TLR-4 in rafts of RAW 264 cells incubated with lipopolysaccharide (LPS) (0.75 to 50 ng/ml) and TNF produced, Pearson’s coefficient = 0.887.

Since signalling complexes formed around CD14 also include TLR-4 [26], the effects of macrophage stimulants upon TLR-4 was studied. Firstly we showed that the amounts of TLR-4 in RAW-264 cells were not significantly altered by the addition of either 50 μM PrP82-146 (100 units ± 5 compared with 98 units ± 7, n = 9, *P* = 0.62) or 50 μM Aβ_42_ (100 units ± 5 compared with 97 units ± 8, n = 9, *P* = 0.49). The formation of signalling complexes involves the recruitment of TLR-4 into specific detergent-resistant membranes commonly called lipid rafts [39,40]. Here we show that the addition of PrP82-146, but not the control peptide (PrP82-146scrambled), caused a dose-dependent increase in the amounts of TLR-4 within (DRMs) rafts (Figure 12A). The amounts of TLR-4 in rafts was also increased after the addition of 50 μM Aβ_1–42_ or αSN, but were not affected by 50 μM Aβ_42–1_ or βSN (Figure 12B). After RAW 264 cells were incubated with different concentrations of PrP82-146, Aβ_1–42_ or αSN there was a significant correlation between the amounts of TLR-4 within rafts and the amounts of TNF produced (Figure 12C). Similar results were obtained when RAW-264 cells were incubated with LPS; namely that LPS-caused TLR-4 to migrate into rafts and that there was a significant correlation between the amounts of TLR-4 in rafts and TNF production (Figure 12D).

### Glimepiride reduces the peptide-induced translocation of TLR-4 into membrane rafts

**Figure 13:**
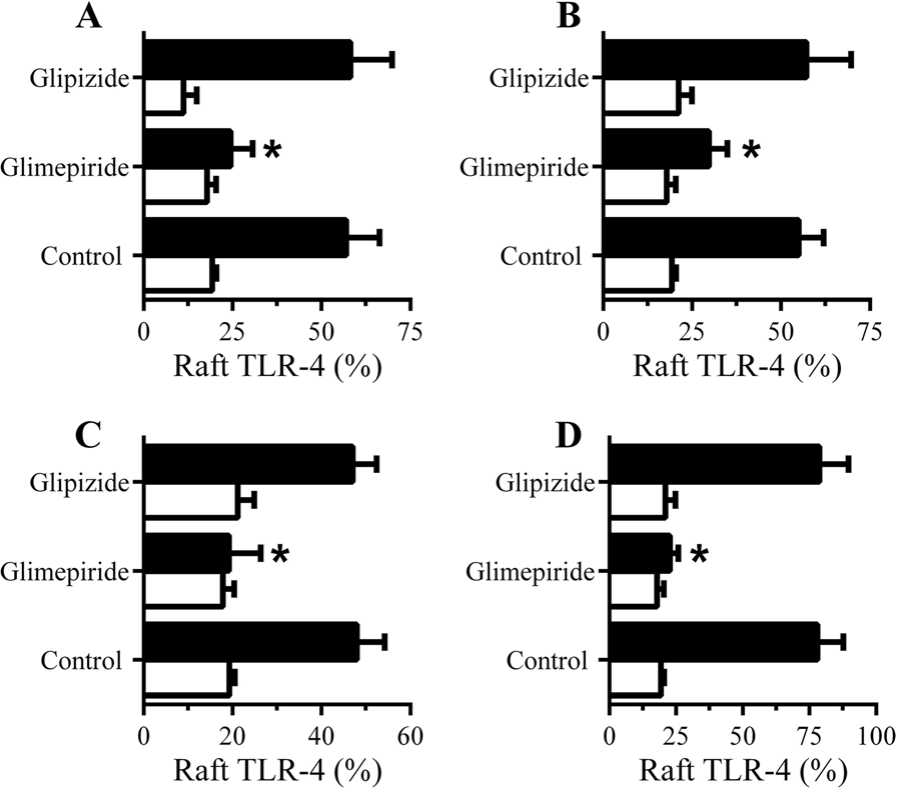
**Glimepiride reduces the translocation of Toll-like receptor (TLR)-4 to rafts. (A)** The % TLR-4 in rafts of RAW 264 cells pre-treated with control medium, 5 μM glimepiride or 5 μM glipizide and incubated with control medium (□) or 50 μM PrP82-146 (■). Values are means ± SD from triplicate experiments performed 4 times, n = 12. *TLR-4 significantly less than those of control cells incubated with PrP82-146. **(B)** The % TLR-4 in rafts of RAW 264 cells pre-treated with control medium, 5 μM glimepiride or 5 μM glipizide and incubated with control medium (□) or 50 μM Aβ_1–42_ (■). Values are means ± SD from triplicate experiments performed 4 times, n = 12. *TLR-4 significantly less than those of control cells incubated with Aβ_1–42_. **(C)** The % TLR-4 in rafts of RAW 264 cells pre-treated with control medium, 5 μM glimepiride or 5 μM glipizide and incubated with control medium (□) or 50 μM αSN (■). Values are means ± SD from triplicate experiments performed 4 times, n = 12. *TLR-4 significantly less than those of control cells incubated with αSN. **(D)** The % TLR-4 in rafts of RAW 264 cells pre-treated with control medium, 5 μM glimepiride or 5 μM glipizide as shown and incubated with control medium (□) or 10 ng/ml LPS (■). Values are means ± SD from triplicate experiments performed 4 times, n = 12. *TLR-4 significantly less than those of control cells incubated with lipopolysaccharide (LPS).

Treatment of RAW264 cells with 5 μM glimepiride did not affect the amounts of TLR-4 either in whole cells (100 units ± 5 compared with 99 units ± 7, n = 9, *P* = 0.71) or within DRMs (rafts) (21 units ± 3 compared with 20 units ± 6, n = 9, *P* = 0.58). Next, we showed that pre-treatment with 5 μM glimepiride reduced the amounts of TLR-4 in rafts in cells incubated with 50 μM PrP82-146 (Figure 13A), 50 μM Aβ_1–42_ (Figure 13B), 50 μM αSN (Figure 13C) or 10 ng/ml LPS (Figure 13D). Pre-treatment of RAW 264 cells with 5 μM glipizide did not affect either peptide or LPS-induced translocation of TLR-4 into DRMs.

## Discussion

There is accumulating evidence that inflammation exacerbates the symptoms and accelerates the progression of AD and PD [10-12]. Consequently, glial cells activated by aggregated proteins are seen as a therapeutic target. Here we show that physiologically relevant concentrations of glimepiride [41] result in the release of several GPI-anchored proteins from RAW 264 and microglial cells consistent with reports that this drug activates an endogenous GPI-PLC [33,34]. Glimepiride treatment of RAW 264 cells also resulted in lower concentrations of cytokine secretion when these cells were incubated with PrP82-146, Aβ_42_ or αSN.

The role of cytokines in neurodegenerative diseases is controversial, although TNF has been reported to protect neurons against Aβ [42,43] the majority of studies suggest that elevated concentrations of cytokines increase neurodegeneration. Thus, elevated levels of TNF are reported in neurodegenerative conditions including AD and PD [44,45], inhibition of TNF prevents neuropathology in mouse models of AD and post-operative cognitive decline [46,47] and the TNF inhibitor etanercept causes cognitive improvement in AD patients [48]. Similarly, high levels of IL-6 are raised in the brain tissue or cerebrospinal fluid (CSF) in AD, AIDS dementia complex, multiple sclerosis, stroke, PD and traumatic brain injuries [49] and IL-6 receptors are expressed on neurons at synapses and can regulate neurotransmitter release [50,51]. As a consequence of these observations it is thought that compounds that reduce cytokine production are likely to reduce neurodegeneration.

As glimepiride would be expected to decrease the expression of many GPI-anchored proteins, including CD14, PrP^C^ and CD55 - proteins which are implicated/involved in cytokine production [25,26,52] - it was not clear which GPI-anchored protein was responsible for cellular responses to aggregated peptides. However we found that CD55 expression was not affected by glimepiride and that antiserum to CD55 did not affect peptide-induced cytokine production. Although glimepiride reduced the PrP^C^ content of RAW 264 cells, cytokine production was not affected by antiserum to PrP^C^. In contrast, antiserum to CD14 blocked peptide- and LPS-induced cytokine production. In addition, microglia from CD14 knockout mice produced fewer cytokines than microglia from CD14 wild type mice when incubated with PrP82-146 or Aβ_1–42_. Collectively, these results indicate that it is the loss of CD14 that is the key element in glimepiride-induced suppression of cytokine production. The loss of CD14 from glimepiride-treated cells was accompanied by a corresponding increase of CD14 in cell supernatants, showing that CD14 was released from cells rather than degraded.

The expression of CD14 is increased in animal models of AD and PD [53] and CD14 is involved in microglial recognition to Aβ fibrils [16] and prion-damaged neurons [24]. Although pre-treatment with glimepiride significantly reduced the amounts of TNF and IL-6 produced by RAW 264 and microglial cells incubated with PrP82-146, αSN or Aβ_42_, it did not reduce the binding of PrP82-146 or Aβ_42_ to RAW 264 cells. Thus, although the presence of soluble CD14 in cell supernatants may bind to PrP or Aβ peptides, such complexes still interacted with cells. Furthermore, cells treated with glimepiride and then washed, so as to remove any soluble CD14, remained hyporesponsive to PrP and Aβ peptides indicating that soluble CD14 alone was not sufficient to explain the immunosuppressive effects of glimepiride. These observations indicate that although cells bind these peptides in a CD14-independent manner, cytokine production is dependent upon the engagement of CD14. Recently, the deletion of CD14 was shown to attenuate AD-like pathology in a transgenic murine model of AD [27] suggesting that CD14-dependent microglial responses to aggregated Aβ are a major driver of the pathology in AD.

Insulin-induced activation of a GPI-PLC generates second messengers that mediate the intracellular effects of insulin [54]. Since glimepiride mimics the effects of insulin upon GPI-PLC [33,55], we hypothesise that it is the glimepiride-induced activation of an endogenous GPI-PLC that leads to the release of CD14 and the suppression of cytokine production. This idea was supported by two observations; firstly, that RAW 264 cells digested with PI-PLC were similar to glimepiride-treated cells in that they contained less CD14 and secreted less cytokines than control cells. Secondly, the inclusion of p-CMPS, a selective inhibitor of GPI-PLC [37], reversed the effects of glimepiride on cellular levels of CD14 and cytokine production.

These observations may have implications beyond neurodegenerative diseases as the pathogenesis associated with many acute infectious diseases may be partly caused following excessive cytokine release by macrophages. For example, LPS is considered to be the major macrophage stimulant leading to excessive cytokine production in gram negative bacterial infections. CD14 was identified as a receptor that mediates LPS-induced cell activation [25] and in some animal models, CD14 knockout mice are resistant to acute septicaemia [56] suggesting that the induction of cytokines via a CD14-dependent mechanism is critical for pathogenesis. Here we show that glimepiride treatment also reduced the LPS-induced secretion of TNF and IL-6. While these results suggest that the loss of CD14 was responsible for the hyporesponsive state of glimepiride-treated macrophages, we noted that glimepiride would be expected to decrease surface expression of other GPI-anchored proteins, and some of these might be responsible for LPS-induced responses. PrP^C^ and CD55 are GPI-anchored proteins which have been implicated in LPS signalling [26]. The predominant role of CD14 in LPS-induced cytokine production in these experiments was demonstrated by both CD14 knockout microglia and neutralising antiserum to CD14.

As glimepiride did not reduce the binding of LPS or neurotoxic peptides to macrophages we investigated cell signalling in these cells. The binding of LPS to CD14 does not initiate cell activation alone; rather it is the first step in the formation of a signalling complex. Thus, CD14 acts as a co-receptor for TLR-4 which mediates activation of myeloid cells [26]. CD14 is targeted to membrane rafts which, in quiescent cells, exist as micro-domains ranging from 5 to 200 nm in diameter [57,58] and which are 'poised’ to form larger rafts [59] and coalesce under specific stimuli to form a larger signalling platform [57,59-62]. The coalescence of individual rafts may bring together receptors and signalling proteins into a functional complex; for example the LPS-induced formation of CD14:MD2:TLR-4 complexes [63]. Notably, this tri-receptor complex is necessary for activation of microglia by Aβ [64] and TLR-4 is associated with increased cytokine production in murine AD models [65,66].

The reorganisation of rafts in the outer membrane leaflet in response to external stimuli can result in the sorting of intracellular signalling molecules on the cytoplasmic leaflet [67-70] and consequently cell activation. For example, extracellular LPS has been shown to recruit the key signalling protein TLR-4 to rafts following engagement of specific receptors [26,63,70] and activate intracellular signalling pathways leading to cytokine production. In this study we demonstrate that PrP82-146, Aβ_1–42_ or αSN also caused the translocation of TLR-4 into rafts and that there was a significant correlation between the amounts of TLR-4 within rafts and cell activation as measured by cytokine production. Critically, this translocation of TLR-4 into rafts was reduced in cells treated with glimepiride.

## Conclusions

Novel approaches of treating neurodegenerative conditions are urgently required. Here we show that glimepiride triggered the release of CD14 from RAW 264 cells consistent with activation of an endogenous GPI-PLC and the shedding of GPI-anchored proteins including CD14 from RAW 264 cells. Consequently, glimepiride treatment significantly reduced cytokine secretion from RAW 264 cells incubated with PrP82-146, Aβ_1–42_ or αSN. Such observations suggest that glimepiride may reduce cytokine secretion and hence neuroinflammation in neurodegenerative diseases and should be considered as a novel adjunctive treatment for AD.

## Abbreviations

AD: Alzheimer’s disease; Aβ: amyloid-β; αSN: α-synuclein; CSF: cerebrospinal fluid; DRM: detergent resistant membrane; EDTA: ethylenediaminetetraacetic acid; ELISA: enzyme-linked-immunoassay; FCS: foetal calf serum; GPI: glycosylphosphatidylinositol; Ig: immunoglobulin; IL: interleukin; LPS: lipopolysaccharide; p-CMPS: p-chloromercuriphenylsulphonate; PBS: phosphate-buffered saline; PD: Parkinson’s disease; PI: phosphatidylinositol; PLC: phospholipase C; PrP^C^: cellular prion protein; SDS: sodium dodecyl sulphate; TLR: Toll-like receptor; TNF: tumour necrosis factor.

## Competing interests

The authors declare that there are no competing interests.

## Authors’ contributions

VI: data collection and analysis and manuscript revision. CB: conception and design, data collection and analysis, manuscript writing and revision. AW: conception and design and manuscript writing and revision. All authors read and approved the final manuscript.
